# Anisotropic Magnetoresistive Sensors: Dynamic Modeling and Characterization for Blade Tip-Timing Measurements

**DOI:** 10.3390/s26082506

**Published:** 2026-04-18

**Authors:** Daniele Busti, Lorenzo Capponi, Antonella Gaspari, Laura Fabbiano, Gianluca Rossi

**Affiliations:** 1 Department of Engineering, University of Perugia, 06125 Perugia, Italy; daniele.busti2@studenti.unipg.it (D.B.); gianluca.rossi@unipg.it (G.R.); 2 Faculty of Mechanical Engineering, University of Ljubljana, 1000 Ljubljana, Slovenia; 3 Department of Mechanics, Mathematics and Management, Polytechnic of Bari, 70125 Bari, Italy; antonella.gaspari@poliba.it (A.G.); laura.fabbiano@poliba.it (L.F.)

**Keywords:** magnetoresistance, dynamics, sensors, tip-timing, turbomachinery

## Abstract

Monitoring of blade vibrations in turbomachinery equipped with ferromagnetic blades is currently performed using the Blade Tip-Timing (BTT) non-contact technique. To reduce measurement uncertainty on time samples, BTT systems require measurement probes to meet high dynamic performance requirements. Anisotropic magnetoresistive (AMR) sensors have recently gained interest for this application owing to their high sensitivity to magnetic flux variations and robustness in harsh, contaminated environments. However, a thorough dynamic characterization of AMR-based BTT probes remains largely unexplored, representing a critical gap in next-generation industrial measurement systems. This work presents a custom-designed signal conditioning circuit tailored for AMR-based BTT measurements, alongside a systematic methodology for characterizing its dynamic performance. The circuit is modeled as a block diagram, from which transfer functions are derived analytically and validated experimentally, providing a rigorous and reproducible framework for probe dynamic assessment. The complete instrumentation chain is then tested on a low-speed rotor test bench in a BTT configuration. Results reveal a fundamental sensitivity–bandwidth trade-off: satisfying the cutoff frequency requirement imposed by BTT applications inherently reduces signal gain below the threshold needed to resolve individual blade-passage events. This finding isolates the key design bottleneck for AMR-based BTT probes and provides quantitative guidance for future optimization of both sensor and circuit design toward industrial tip-timing deployment.

## 1. Introduction

Vibration-induced high cycle fatigue is among the leading causes of failure in industrial turbomachinery, such as large-scale steam turbines [1], heavy-duty industrial compressors, and early stages of industrial gas turbine, making blade vibration monitoring an essential requirement for both design certification and in-service health assessment [2]. Although strain gauges [3] provide direct stress measurements, they are invasive, limited only to the instrumented blades, and require complex telemetry systems while remaining vulnerable to extreme thermal and centrifugal loads [3]. The non-contact Blade Tip-Timing (BTT) technique overcomes these drawbacks by inferring blade deflection from blade tip time-of-arrival measurements at casing-mounted proximity sensors, enabling non-invasive, telemetry-free monitoring of all blades [4,5]. Nevertheless, metrological validation and uncertainty estimation remain open challenges, and innovative measurement systems are still needed to bring BTT certification reliability on par with established methods [6,7]. Several non-contact sensing technologies have been investigated for BTT measurements, including eddy current probes, capacitive sensors, and microwave-based systems [8]. Among these, optical sensors are among the most widely adopted solutions [9], valued for their excellent spatial resolution and fast signal rise times [8]. However, their performance degrades significantly in the harsh environments typical of operating turbomachinery, where oil, moisture, and particulate contamination can rapidly obscure optical surfaces and compromise long-term reliability [10].

In such environments, magnetic field-based sensors offer a robust alternative. Back-biased Hall effect sensors have historically been the standard for detecting rotating metallic targets [11], though their inherently low SNR limits measurement sensitivity [12]. This has driven an industry-wide transition toward the anisotropic magnetoresistance (AMR) principle. AMR sensors exploit the dependence of electrical resistance in thin ferromagnetic films on the angle between local magnetization and current flow, making them highly sensitive to the magnetic flux variations induced by passing ferromagnetic targets [13]. Compared to Hall effect ones, AMR sensors provide a significantly higher SNR and operate across wider air gaps, translating the magnetic field modulation induced by passing blades into electrical pulses. Their solid-state construction, low power consumption, and minimal signal drift further support integration into compact, modular monitoring systems suitable for industrial deployment [14,15].

In recent years, AMR sensors have been explored for Blade Tip-Timing (BTT) applications, demonstrating their potential as a robust alternative to optical probes. In 2011, Procházka and Vaněk [16] developed the VDS-UT system. Leveraging MR technology, this approach enabled the online monitoring of blade vibration amplitudes and frequencies, making it possible to evaluate not only their static position but also complex phenomena such as elongation and untwisting. The investigation into the potential of these sensors was expanded starting in 2013 by Procházka et al. [17,18], who compared different contactless measurement principles, focusing on the development of new types of magnetoresistive sensors specifically designed to operate inside steam turbines, including configurations with shrouded blades. The application of magnetoresistive sensors was extended to the simultaneous measurement of multiple parameters. Between 2015 and 2016, Tomassini et al. [19,20] introduced and tested magnetoresistive probes capable of jointly performing tip-timing and tip-clearance, ensuring measurement uncertainties in line with industrial standards while maintaining low manufacturing costs. The accuracy of these sensing architectures was then the subject of further studies: in 2022, Tchuisseu et al. [15] proposed methodologies to optimize the physical positioning of MR probes, thereby improving the overall performance and reliability of monitoring systems. More recently, research attention has strongly focused on the experimental validation and dynamic behavior of AMR sensors. In 2024, Procházka et al. [21] conducted an extensive experimental campaign dedicated to the performance of AMR sensors. Continuing in this direction, in 2026, Mekhalfia et al. [22] thoroughly analyzed the influence of the blade angle and clearance on the voltage response of the AMR sensor, providing crucial insights for the interpretation and optimization of signals under real operating conditions. Despite these advancements [15,16,17,18,19,20,21,22], the dynamic characterization of AMR sensors for BTT applications remains an open challenge: no prior work has systematically modeled or experimentally validated the dynamic response of an AMR-based probe and its associated signal conditioning electronics in this context. At the tangential speeds characteristic of turbomachinery, the sensor must resolve extremely brief blade-passage events, making bandwidth a fundamental yet so far unquantified performance requirement for this class of probes [23].

This work addresses this gap through three main contributions. First, a custom signal conditioning circuit is designed and purpose-built for AMR-based BTT measurements. Second, an analytical model of the complete instrumentation chain is developed as a transfer function framework derived from a block diagram representation of the circuit, and rigorously validated through experimental measurements, providing a systematic and reproducible methodology for AMR probe dynamic characterization. The system was validated on a low-speed test bench as a proof-of-concept currently intended for laboratory environments. Adapting this architecture to operate in high-speed and high-temperature [24] conditions represents the goal of future developments. Together, these contributions yield quantitative insight into the fundamental sensitivity–bandwidth trade-off governing AMR-based BTT probe design and establish a modeling framework to guide future optimization toward industrial deployment.

The manuscript is organized as follows. Section 2 provides a theoretical background on magnetoresistance principles and Wheatstone bridge operation. Section 3 describes the methodology for sensor and signal condition circuit design and characterization, while Section 4 presents the experimental setup. Section 5 presents the dynamic characterization results, and Section 6 draws the conclusions.

## 2. Theoretical Background

### Magnetoresistance

In 1856, William Thomson first observed that the electrical resistance of iron varies upon magnetization [25]. This phenomenon, known as the magnetoresistive effect, denotes the change in electrical resistivity of a material induced by an external magnetic field [26]. Although universal, its magnitude varies markedly across materials: in most metals, the effect amounts to a fraction of a percent and is therefore negligible for practical purposes, while in select ferromagnetic materials, it can reach several percent, making it of practical significance for sensing applications [27].

**Figure 1:**
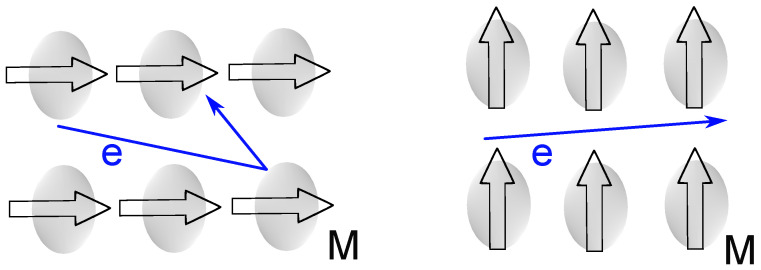
Schematic illustration of the AMR effect: distortion of electron orbitals gives rise to anisotropic scattering when the macroscopic magnetization (M) is (**left**) parallel and (**right**) perpendicular to the current direction. The blue arrows (e) represent the trajectories of conduction electrons.

Among the various magnetoresistive mechanisms [28,29,30], a particularly well-established one is anisotropic magnetoresistance (AMR), in which the resistivity of a material depends on the relative orientation between the electrical current and the internal magnetization vector [31]. The physical origin of AMR lies in spin–orbit coupling, which causes the electron orbitals to deform in response to the magnetization direction, leading to anisotropic conduction electron scattering (see Figure 1).

The resistance *R* of an AMR element can be expressed as a function of the angle α between the current direction and the magnetization vector as [31]

(1) $$R\left(\alpha \right)=R_{0}+\Delta R^{\prime }\cos^{2}\alpha ,$$

where $\Delta R^{\prime }$ denotes the maximum resistance variation in the sensing element and $R_{0}$ is its minimum resistance. When an external magnetic field is applied, the magnetic domains within the material tend to align with the field, modifying the orientation of the net magnetization and, consequently, the angle α.

**Figure 2:**
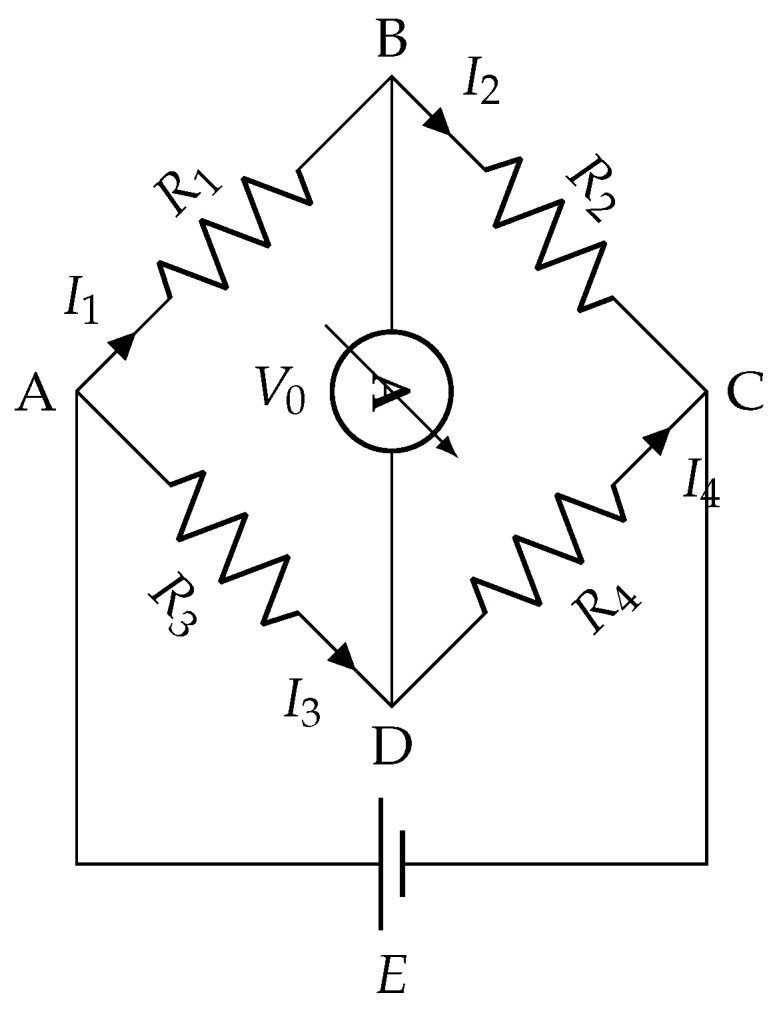
Schematic of a Wheatstone bridge circuit.

The change in resistance described by (1) is typically small, and measuring it accurately against a large baseline resistance $R_{0}$ is challenging with a simple two-terminal configuration. The Wheatstone bridge circuit addresses this by converting the differential resistance variation into a measurable output voltage, effectively rejecting the common-mode baseline and amplifying the sensitivity to small changes [32]. In its general form, the bridge comprises four resistors, a galvanometer, and a DC voltage source, as shown in Figure 2.

Applying Kirchhoff’s voltage law to each independent loop of the circuit in Figure 2 yields the following system of equations [33]:

(2) $$\begin{array}{c} \left\{\begin{array}{c} E-V_{DC}-V_{AD}=0\\ E-V_{AB}-V_{BC}=0\\ V_{0}-V_{BC}+V_{DC}=0 \end{array}\right.\;⟹\;\left\{\begin{array}{c} E=\left(R_{4}+R_{3}\right)\;I_{3}\\ E=\left(R_{1}+R_{2}\right)\;I_{2}\\ V_{0}=V_{BC}-V_{DC}. \end{array}\right. \end{array}$$

Substituting the expressions for $I_{2}$ and $I_{3}$ from Equation (2) into the third equation and applying Ohm’s law gives the bridge output voltage as a function of the resistances and supply voltage [34]:

(3) $$V_{0}=R_{2}\cdot I_{2}-R_{4}\cdot I_{3}=\frac{R_{2}\;E}{R_{1}+R_{2}}-\frac{R_{4}\;E}{R_{4}+R_{3}}=\left(\frac{R_{2}}{R_{1}+R_{2}}-\frac{R_{4}}{R_{4}+R_{3}}\right)E.$$

The bridge is balanced when $V_{0}=0$, which requires

(4) $$\frac{R_{2}}{R_{1}+R_{2}}=\frac{R_{4}}{R_{4}+R_{3}}\;⟹\;R_{2}R_{3}=R_{1}R_{4}.$$

**Figure 3:**
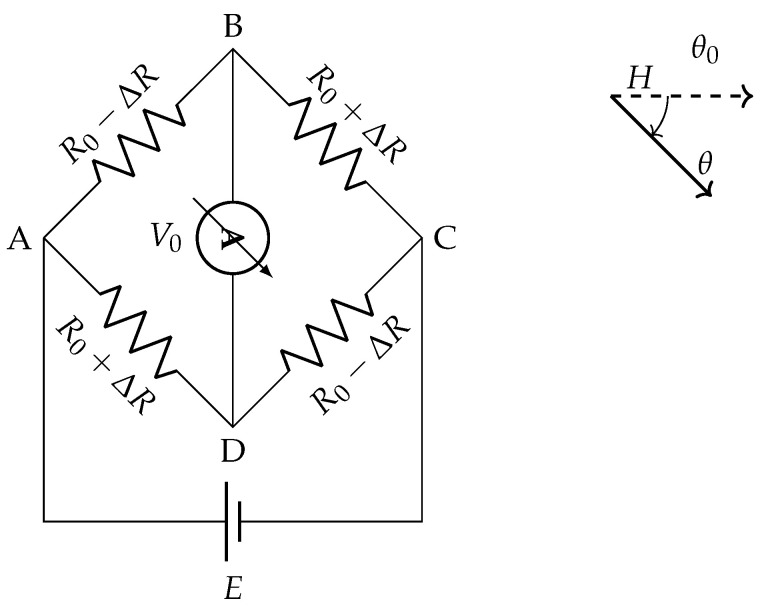
Resistance variations in the AMR sensing elements for a field rotation θ from the nominal direction $\theta_{0}$.

The advantage of using AMR elements in a Wheatstone bridge becomes clear when the sensing elements are oriented to respond differentially to an applied field. Figure 3 shows the configuration adopted in a typical AMR sensor, in which the four sensing elements are arranged such that a rotation θ of the external field causes $R_{1}$ and $R_{4}$ to increase while $R_{2}$ and $R_{3}$ decrease by the same amount (or vice versa), maximizing the bridge imbalance and hence the output sensitivity [35]. A saturating bias field is applied along the nominal direction $\theta_{0}$ to ensure single-domain alignment of all elements prior to any field rotation.

The resistances $R_{2}$ and $R_{3}$ vary with θ according to

(5) $$R_{2}=R_{3}=R_{0}+\Delta R^{\prime }\cos^{2}\;\left(-\frac{\pi }{4}+\theta \right)=R_{0}+\frac{\Delta R^{\prime }}{2}\left(1+\sin2\theta \right),$$

while $R_{1}$ and $R_{4}$ follow the complementary law:

(6) $$R_{1}=R_{4}=R_{0}+\Delta R^{\prime }\cos^{2}\;\left(\frac{\pi }{4}+\theta \right)=R_{0}+\frac{\Delta R^{\prime }}{2}\left(1-\sin2\theta \right).$$

**Figure 4:**
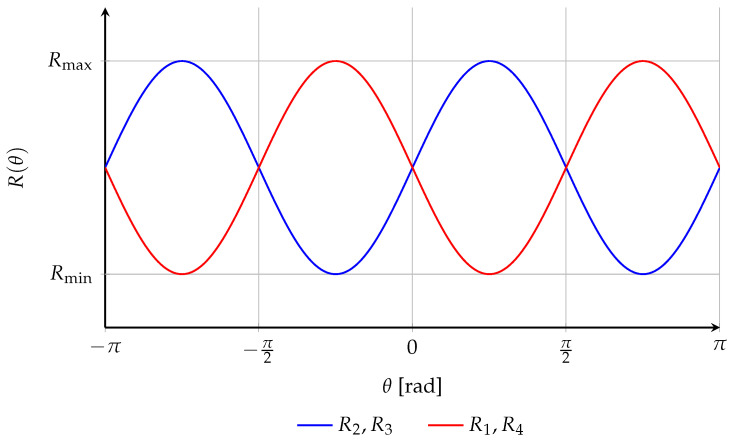
Angular dependence of the resistance pairs $R_{1,4}$ and $R_{2,3}$ as a function of the applied field angle θ.

The angular dependence of both resistance pairs is illustrated in Figure 4.

Substituting Equations (5) and (6) into Equation (3), the sensor output voltage simplifies to

(7) $$V_{0}=\frac{\Delta R^{\prime }}{2R_{\mathrm{med}}}\;\sin\left(2\theta \right)\;E,$$

where $R_{\mathrm{med}}=\left(R_{\max}+R_{\min}\right)/2$ is the mean resistance. The Wheatstone bridge configuration thus transduces the rotation θ of the applied field into an output voltage. The AMR elements are oriented such that any change in the magnetization direction increases one resistance pair while simultaneously decreasing the other, maintaining $R_{1}=R_{4}$ and $R_{2}=R_{3}$, thereby unbalancing the bridge and generating a differential output signal, as illustrated in Figure 4.

## 3. Materials and Methods

In this section, the design and characterization of the sensing element and signal conditioning circuit are described. The sensing element is selected based on its suitability for BTT applications, while the conditioning circuit is custom-designed to meet the specific requirements of AMR-based measurements. The dynamic performance of the complete instrumentation chain is then analyzed through a combination of analytical modeling and experimental validation.

### 3.1. Sensing Element

Following a comparative evaluation of available sensing solutions, the Honeywell HMC1501-TR (Honeywell International Inc., Morristown, NJ, USA) was chosen for this work, owing to its robust AMR response, high signal-to-noise ratio, and suitability for operation under saturated magnetic field conditions. Furthermore, according to its technical specifications, the sensor is designed for an operating temperature range of −40 °C to +125 °C. This range is unsuitable for in-service turbomachinery, but fully applicable to laboratory testing. Its sensing element consists of a Wheatstone bridge of AMR resistors whose resistance varies with the applied field direction. According to its specifications, the sensor features a typical bridge resistance of 5000Ω. When the applied field exceeds the saturation threshold of 5 mT, the thin-film magnetization aligns with the field, making the sensor responsive exclusively to angular variations rather than field magnitude. Consistent with Equation (7), the differential output voltage under these conditions is [35]:

(8) $$V_{0}=V_{b}\cdot S\cdot \sin\left(2\theta \right),$$

where $V_{b}$ is the bridge supply voltage and S=12 mV/V is the nominal sensitivity.

A key figure of merit for magnetoresistive devices is the MR ratio:

(9) $$MR=\frac{R_{\max}-R_{\min}}{R_{\min}}\times 100\%.$$

AMR sensors typically exhibit MR ratios of 1–2% [31], directly constraining the signal amplitude available for BTT measurements. By rewriting Equation (7) as a function of the MR ratios described in Equation (9), the nominal sensitivity *S* introduced in Equation (8) can be explicitly defined. The output voltage becomes

(10) $$V_{0}=\frac{MR}{MR+2}V_{b}\sin\left(2\theta \right)$$

This formulation highlights that the output voltage amplitude is directly proportional to the MR ratio. Based on the typical peak-to-peak output voltage of 120mV at $V_{b}=5\;V$ reported in the datasheet of the sensing element, a nominal MR ratio of approximately 2.43% can be derived using Equation (10). Given the intrinsically low MR values of AMR sensors, the resulting BTT signal transients are severely constrained in amplitude. Consequently, a dedicated signal conditioning circuit featuring an operational amplifier is required to suitably amplify the sensor output for BTT measurements.

### 3.2. Signal Conditioning Circuit

**Figure 5:**
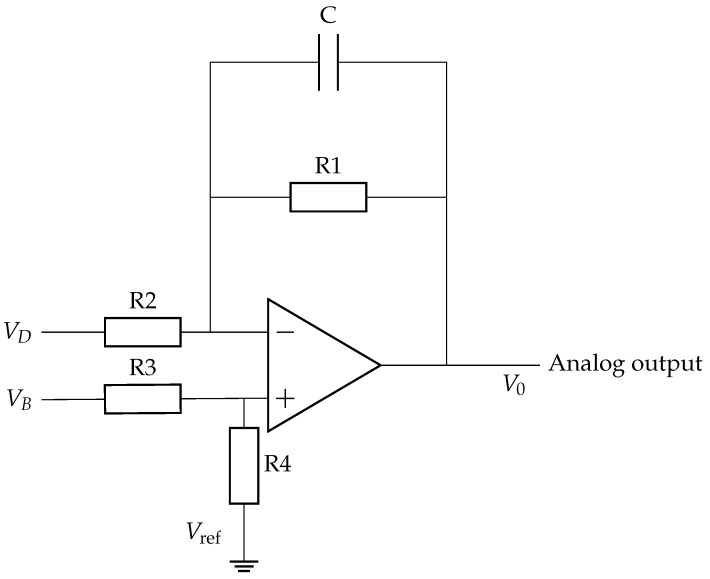
Schematic of the signal conditioning circuit.

The small signal amplitude imposed by the low MR ratio places stringent requirements on the conditioning circuit, which must provide sufficient gain while preserving the bandwidth needed to resolve blade-passage events. The circuit employs a high-speed OPA2356A (Texas Instruments, Dallas, TX, USA) operational amplifier, featuring a 200 MHz unity-gain bandwidth and powered by a 3 V supply voltage, in a differential configuration to convert the bridge imbalance voltage into a single-ended output suitable for acquisition. The conditioning circuit, shown in Figure 5, comprises a differential amplifier with a parallel RC feedback network and input resistors at both bridge terminals.

Applying the ideal op-amp model and the superposition principle, the output voltage is derived as

(11) $$V_{0}=V_{\mathrm{ref}}+\frac{R_{1}}{R_{2}\;\left(1+j\omega \;R_{1}C\right)}\;\left(V_{B}-V_{D}\right),$$

where $V_{B}-V_{D}$ is the differential bridge voltage. The transfer function exhibits a low-pass characteristic with DC gain $R_{1}/R_{2}$ and cutoff frequency

(12) $$f_{c}=\frac{1}{2\pi R_{1}C}.$$

The values of $R_{1}$, $R_{2}$, and *C* are selected to provide adequate gain to resolve the millivolt-level sensor output while maintaining a measurement bandwidth sufficient for the blade-passage frequencies of interest.

### 3.3. Frequency Response Analysis

To isolate the dynamic contribution of the conditioning circuit, the sensing element is treated as quasi-static. The AMR Wheatstone bridge consists entirely of passive resistive elements, which introduce no significant poles or zeros within the operational frequency range; the dynamic behavior of the system is therefore attributed solely to the active conditioning stage.

#### 3.3.1. RC Feedback Network

Under the ideal op-amp assumption (infinite open-loop gain and unlimited bandwidth), the frequency response of the conditioning stage is governed exclusively by the passive RC feedback network, yielding a first-order low-pass transfer function:

(13) $$H\left(j\omega \right)=\frac{1}{1+j\omega R_{1}C},$$

with cutoff frequency $f_{c}=1/\left(2\pi R_{1}C\right)$, set by the time constant $\tau =R_{1}C$ of the feedback network.

#### 3.3.2. Operational Amplifier

**Figure 6:**
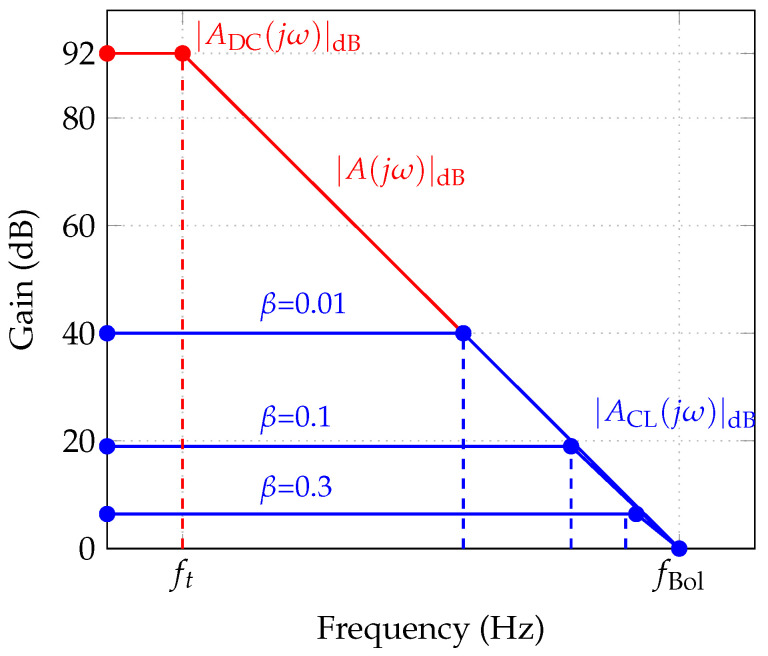
Bode magnitude plots illustrating the gain–bandwidth trade-off for the operational amplifier under varying feedback factors β. Increasing β reduces the closed-loop DC gain while extending the −3dB bandwidth proportionally.

In practice, the open-loop gain of a real op-amp is frequency-dependent and is modeled as a single-pole system:

(14) $$A\left(j\omega \right)=\frac{A_{0}}{1+j\frac{\omega }{\omega_{0}}},$$

where $A_{0}$ is the DC open-loop gain and $\omega_{0}$ is the open-loop pole frequency. Applying KCL at the amplifier terminals under the symmetry condition $R_{1}=R_{4}$ and $R_{2}=R_{3}$, and defining the feedback factor $\beta =R_{2}/\left(R_{1}+R_{2}\right)$ and the input attenuation $\alpha =R_{1}/\left(R_{1}+R_{2}\right)$, the closed-loop gain takes the form

(15) $$A_{\mathrm{CL}}\left(j\omega \right)=\frac{\alpha A_{\mathrm{CL}0}}{1+j\frac{\omega }{\omega_{\mathrm{CL}0}}},$$

where $A_{\mathrm{CL}0}=A_{0}/\left(1+A_{0}\beta \right)$ is the closed-loop DC gain and $\omega_{\mathrm{CL}0}=\omega_{0}\left(1+A_{0}\beta \right)$ is the feedback-extended bandwidth. As shown in Figure 6, negative feedback reduces the DC gain while proportionally extending the bandwidth, a fundamental trade-off governed by the feedback factor β.

#### 3.3.3. Combined Circuit Dynamics

The analyses above treated the dynamics of the RC feedback network and of the op-amp separately, in order to clarify their individual contributions. In the complete circuit of Figure 5, however, these two mechanisms act simultaneously and jointly determine the closed-loop frequency response. Moreover, the impedance asymmetry between the RC feedback branch and the reference branch invalidates a purely differential description: $V_{0}$ can no longer be expressed as a single transfer function H(jω) acting on $V_{B}-V_{D}$ alone. The system must therefore be formulated as a Multi-Input Single-Output (MISO) system with independent inputs $V_{B}$ and $V_{D}$, so that the combined effects of the feedback network and op-amp dynamics can be captured within a unified closed-loop model:

(16) $$V_{0}=A\left(\frac{R_{4}}{R_{4}+R_{3}}V_{B}+\frac{R_{3}}{R_{4}+R_{3}}V_{\mathrm{ref}}-\frac{R_{1}}{R_{1}+R_{2}\left(1+j\omega R_{1}C\right)}V_{D}-\frac{R_{2}\left(1+j\omega R_{1}C\right)}{R_{1}+R_{2}\left(1+j\omega R_{1}C\right)}V_{0}\right)$$

**Figure 7:**
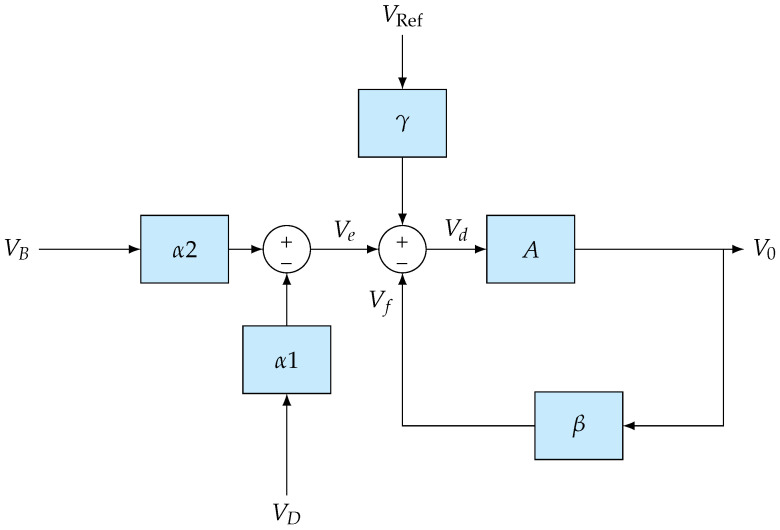
Block diagram of the signal conditioning system.

Under the symmetry condition $R_{1}=R_{4}$, $R_{2}=R_{3}$, the system structure is captured by the block diagram in Figure 7, with network parameters:

(17) $$\beta =\frac{R_{2}\left(1+j\omega R_{1}C\right)}{R_{1}+R_{2}\left(1+j\omega R_{1}C\right)},\;\alpha_{1}=\frac{R_{1}}{R_{1}+R_{2}\left(1+j\omega R_{1}C\right)},\;\alpha_{2}=\frac{R_{4}}{R_{4}+R_{3}}.$$

The two resulting closed-loop transfer functions are:

(18) $$A_{\mathrm{CL},B}=\frac{A\;\alpha_{2}}{1+A\beta },\;A_{\mathrm{CL},D}=\frac{A\;\alpha_{1}}{1+A\beta }$$

To identify the governing dynamics, both closed-loop transfer functions of the MISO system were simulated in MATLAB (version 20020b) using actual component values. The analysis shows that the two paths exhibit markedly different time constants. The path characterized by the dominant pole (the one closest to the origin in the *s*-plane) presents a significantly longer transient response and is hereafter defined as the *slow channel*. Given that this channel acts as the primary limiting factor for the overall settling time, the system dynamics are effectively dictated by its behavior. For practical characterization purposes, this simplification reduces the MISO system to an equivalent single-input description, safely neglecting the faster channel’s contributions, which decay earlier.

#### 3.3.4. Transfer Function Estimation

The transfer function H(f) was estimated from simultaneously acquired input $V_{in}\left(t\right)$ and output $V_{0}\left(t\right)$ signals—phase-locked acquisition being required to preserve the cross-spectral phase—using the $H_{1}$ spectral estimator [36]. For a linear time-invariant system, the input–output relationship in the frequency domain is:

(19) Y(f)=H(f)·X(f).

Multiplying both sides by the complex conjugate of the input spectrum and taking the ensemble average yields:

(20) $$H\left(f\right)=\frac{S_{YX}\left(f\right)}{S_{XX}\left(f\right)},$$

where $S_{YX}\left(f\right)$ is the cross-power spectral density (CPSD) between output and input, and $S_{XX}\left(f\right)$ is the auto-power spectral density (PSD) of the input. The $H_{1}$ estimator minimizes the effect of additive noise on the output side under the assumption that the input signal is noise-free, making it well-suited to bench characterization where the excitation is a controlled, low-noise source. In this study, both spectral quantities were computed in MATLAB using the the cpsd and pwelch functions. A Hann window was selected to minimize spectral leakage while maintaining a good compromise with the main-lobe width. A segment length of N=2048 samples was chosen as an optimal trade-off between achieving an adequate frequency resolution and retaining a sufficient number of segments for ensemble averaging, which is crucial to reduce the estimator variance. A 50% overlap was applied to compensate for the data attenuation at the edges of the Hann window, maximizing data utilization. Finally, the sampling rate of $f_{s}=125\;\mathrm{MS}/s$ was established by the acquisition hardware capabilities to strictly satisfy the Nyquist criterion and prevent aliasing. Consequently, the frequency resolution of the transfer function is calculated as $\Delta f=f_{s}/N=125\times 10^{6}/2048\approx 61.035\;\mathrm{kHz}$.

## 4. Experimental Campaign

### 4.1. Signal Conditioning Circuit Characterization

The signal conditioning circuit was implemented around the OPA2356AIDR [37], a high-speed operational amplifier from Texas Instruments. The device was selected for its 200 MHz unity-gain bandwidth, which ensures the target closed-loop gain is maintained across the frequency range relevant to turbine blade vibration measurements. The amplifier is operated in single-supply mode at $V_{CC}=3$ V, with the non-inverting input biased to a mid-supply reference. The operational amplifier selection was guided by three concurrent requirements: achieving the target closed-loop gain, maintaining a sufficiently wide bandwidth for fast blade-passage signals, and ensuring adequate phase margin in the presence of parasitic capacitances inherent to the PCB layout, cabling, and the amplifier input.

The inverting input is driven through an input resistance of $R_{2}=2.2\;k\Omega$, chosen to prevent loading of the AWG source. To investigate the circuit’s performance under different closed-loop gains, three distinct setups were prepared by varying the feedback resistance: 4.6kΩ, 100kΩ and 680kΩ. In each configuration, the feedback resistor is placed in parallel with a 1pF capacitor. The chosen feedback resistance dictates the DC closed-loop gain, while the parallel capacitor serves exclusively as a stability compensator. Parasitic capacitances at the inverting input reduce the noise gain at high frequencies, decreasing the rate of closure between the open-loop gain and the noise gain 1/β on the Bode plot and eroding the phase margin. The parallel feedback capacitor introduces a compensating zero that partially restores the phase margin and stabilizes the high-frequency response across the different setups.

**Figure 8:**
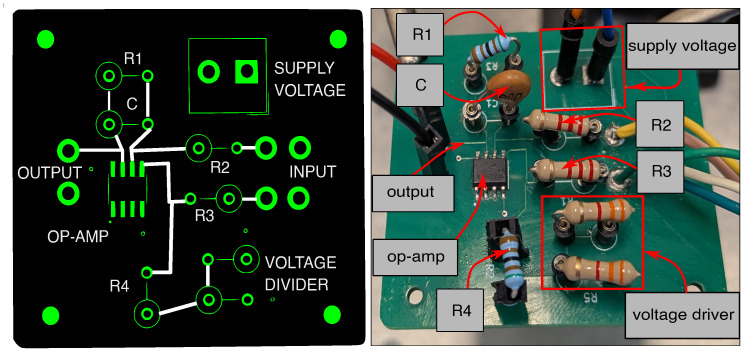
Custom PCB implementation of the signal conditioning circuit.

The circuit was realized on a custom PCB, shown in Figure 8, integrating the amplifier and all passive components required for signal conditioning.

The frequency response was characterized experimentally using a PicoScope 5444B (Pico Technology, Cambridgeshire, UK), which integrates a high-resolution digital oscilloscope with an Arbitrary Waveform Generator (AWG). The AWG generated a logarithmic sinusoidal sweep spanning 1 Hz to 20 MHz over a duration of 40 ms. For each distinct configuration of the conditioning circuit, the amplitude of the input signal was adjusted to 50 mV, 20 mV, and 2 mV, applied directly to the inverting input. The amplitude was varied across configurations to maintain the amplifier output within its linear range (supply voltage 3V); a constant input amplitude would cause output saturation at higher gain settings. Since the $H_{1}$ estimator computes $H\left(f\right)=S_{YX}\left(f\right)/S_{XX}\left(f\right)$, a quantity that is independent of excitation amplitude for a linear system, varying the input level does not affect the validity or comparability of the frequency-response measurements. The input signal was simultaneously monitored via a BNC T-splitter, with one branch connected to the circuit under test and the other routed to a dedicated oscilloscope channel, ensuring phase-coherent acquisition of both signals.

Data were acquired at a sampling rate of $f_{s}=125\;\mathrm{MS}/s$, corresponding to a Nyquist frequency of 62.5MHz. This provides an oversampling ratio of approximately 6× relative to the maximum sweep frequency of 20 MHz, safely preventing aliasing. The 500 ms acquisition window fully encompassed the sweep duration.

### 4.2. BTT Measurement Test Case

**Figure 9:**
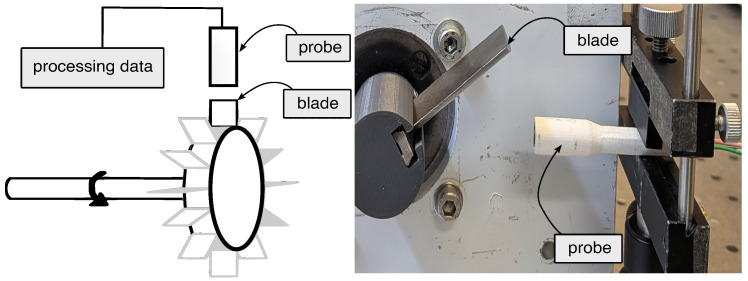
Schematic of the BTT test bench: DC motor, ferromagnetic blade target, AMR probe, and signal conditioning circuit.

To validate experimentally the signal conditioning circuit and the developed model, a dedicated probe-development test bench was built (Figure 9).

**Figure 10:**
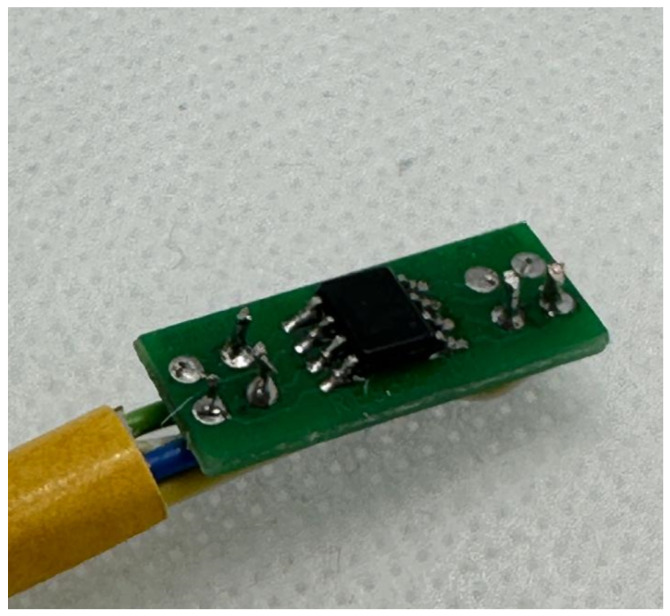
HMC1501-TR magnetoresistive sensor assembled on the custom PCB.

A custom PCB was designed in KiCad 9.0 to interface the HMC1501-TR AMR sensor (Honeywell, Morristown, NJ, USA), housed within a 3D-printed cylindrical casing, with the signal conditioning circuit, powered at 3 V. Sensor connections are routed to plated through-holes (PTHs) to allow flexible external wiring during prototyping. The assembled sensor PCB is shown in Figure 10.

The measurement target consists of a single ferromagnetic blade driven by a 1.2V DC motor. The AMR probe is rigidly mounted on a fixed radial support, with its sensing face aligned perpendicular to the blade path, maintaining a nominal air gap of 3mm [±0.1mm] between the probe face and the blade tip. The blade is made of ferromagnetic stainless steel (AISI 410), with a thickness at the root of 7.5 mm, a chord length of 30 mm, and a height of 80 mm. Seven cubic permanent magnets (5mm side) are fixed at the rear of the probe housing, 10mm from the sensor face arranged in a linear row. This arrangement establishes a stable bias magnetic field that is periodically perturbed by the passing ferrous blade: each blade passage modifies the reluctance of the magnetic circuit, altering the net field direction at the sensor and generating a measurable output transient. The output signal was acquired with a PicoScope 5444B (Pico Technology, Cambridgeshire, UK) at a sampling rate of 5 MS/s—providing a Nyquist frequency well above the expected blade-passage harmonics—over a 50 s acquisition window. AC coupling was selected to suppress the DC bias offset inherent to the bridge output, and the input range was set to ±50 mV to match the expected signal amplitude. It should be noted that the rotational speeds employed in this test bench (83–750 rpm) are significantly lower than those encountered in industrial BTT applications; however, this does not affect the validity of the findings, as the scope of the experimental campaign is limited to the validation of the sensor and signal conditioning circuit model, rather than the characterization of high-speed blade dynamics.

## 5. Results and Discussion

Results are presented in two parts. First, the frequency response of the conditioning circuit is characterized experimentally and compared to theoretical predictions. Second, the blade-passage signals acquired under different gain configurations are analyses to assess the detectability of blade-transit events and to quantify the gain–bandwidth trade-off inherent in the system.

### 5.1. Signal Conditioning Circuit Characterization

**Figure 11:**
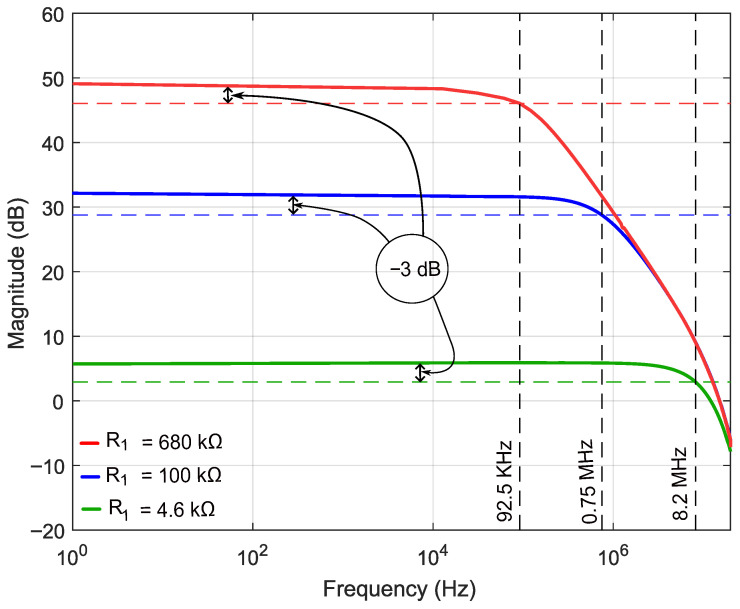
Experimentally obtained Bode magnitude plot of the conditioning circuit closed-loop transfer function ($R_{2}=R_{3}=2.2\;k\Omega$, C=1pF).

The conditioning circuit was characterized by measuring the closed-loop transfer function of the complete system, as derived in Section 3.3.3, associated with the slower of the two input dynamics and therefore representing the dominant bandwidth bottleneck of the overall response. The experimentally obtained Bode magnitude plots for the three feedback resistance configurations are shown in Figure 11. Specifically, a DC gain of ≈2.1× corresponds to a measured −3dB cutoff frequency of 8.2MHz; a gain of ≈45× yields a measured cutoff of 0.75MHz; and the highest gain configuration of ≈309× corresponds to a measured cutoff of 92.5kHz, as summarized in Table 1.

Frequencies are consistently lower than the theoretical predictions, as summarized in Table 1. The discrepancies range from a factor of approximately 2× at moderate gain to more than 4× at the lowest feedback resistance.

These systematic deviations are attributed to parasitic impedances that are negligible at low frequencies but become dominant approaching the theoretical cutoff. Likely contributors include PCB trace capacitance, stray capacitance at the amplifier input nodes, and the finite input capacitance of the oscilloscope probe. A secondary contribution arises from the finite bandwidth of the acquisition system itself. It should be noted that the individual contributions of these parasitic elements were not directly characterized in this work; their combined effect was inferred from the observed frequency discrepancies. A dedicated impedance-analyzer measurement or layout-extracted SPICE simulation to quantify each contribution individually is identified as a direction for future work. Despite these discrepancies, the analytical model developed in Section 3.3.3 proved to be a valuable design tool. By identifying the circuit as a Multiple-Input Single-Output (MISO) system, it established the measurement methodology required to characterize the closed-loop dynamics and provided reliable a priori estimates of the cutoff frequencies via Equation (18). Most importantly, the model elucidates the inherent gain–bandwidth trade-off, which directly governs the sensor performance in BTT applications, as discussed in the following subsection. Notwithstanding the parasitic effects, the 4.6kΩ configuration achieves a measured cutoff of 8.2MHz, which is fully sufficient to resolve the blade-passage frequencies of interest under nominal operating conditions.

### 5.2. BTT Measurement Test Case

The three conditioning circuit configurations were evaluated in a controlled blade-passage test to characterize the gain–bandwidth trade-off and to identify the minimum gain required for reliable blade-transit detection under the available magnetic bias conditions.

**Figure 12:**
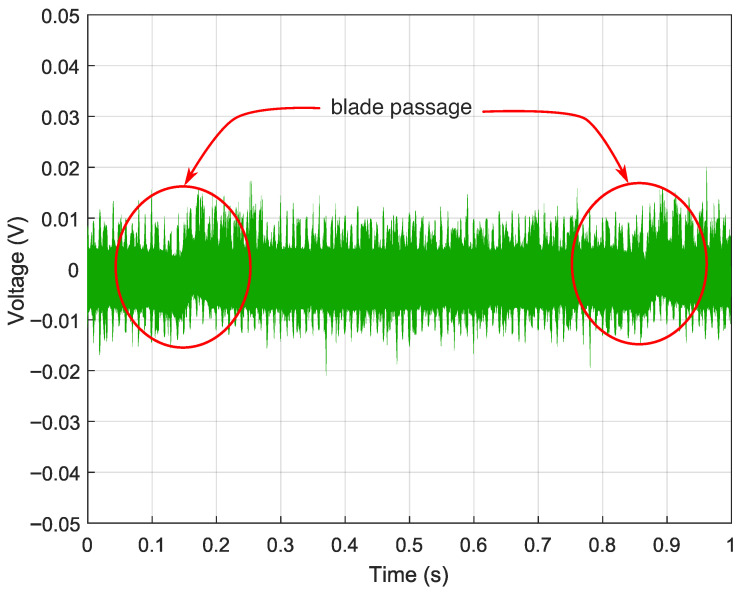
Acquired output signal with low-gain configuration ($R_{2}=R_{3}=2.2\;k\Omega$, $R_{1}=R_{4}=4.6\;k\Omega$, C=1pF, gain ≈2.1×): blade-passage modulation is buried in noise (SNR<1).

Figure 12 shows the acquired output waveform for the nominal gain configuration ($R_{1}=R_{4}=4.6\;k\Omega$). No blade-passage modulation is discernible: the signal is dominated by background noise, indicating that the magnetic perturbation induced by the blade lies below the noise floor (SNR<1). Although this configuration provides bandwidth well in excess of operational BTT requirements, the signal amplitude is insufficient for reliable detection. This is a consequence of the combined effect of the low MR ratio of the sensing element (≈2.43%), the bias field intensity established by the permanent magnet arrangement, and the blade-induced field perturbation at the 3mm air gap; all of these factors compound to reduce the sensor output below the detectable threshold at this gain level.

**Figure 13:**
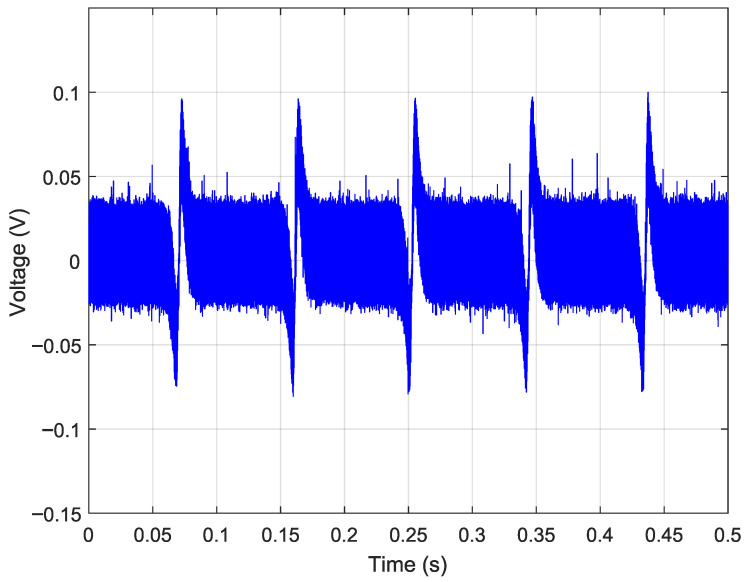
Acquired output signal with medium-gain configuration ($R_{2}=R_{3}=2.2\;k\Omega$, $R_{1}=R_{4}=100\;k\Omega$, C=1pF, gain ≈45×): blade-passage events are detectable with reduced bandwidth.

Increasing the feedback resistance to $R_{1}=R_{4}=100\;k\Omega$ raises the voltage gain to approximately 45× (Equation (18), evaluated at ω=0). As shown in Figure 13, blade-passage events become clearly observable at this gain level. However, the corresponding bandwidth is reduced to 0.75MHz, and the dynamic range is partially compressed due to the higher amplification.

**Figure 14:**
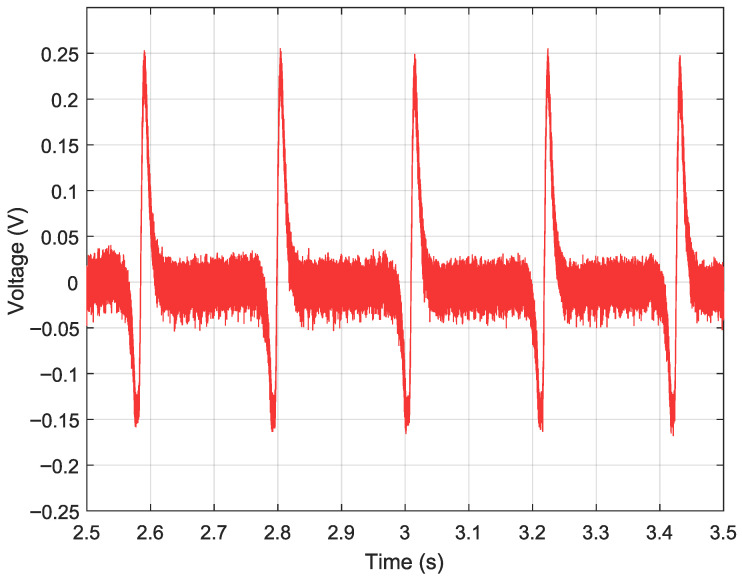
Acquired output signal with high-gain configuration ($R_{2}=R_{3}=2.2\;k\Omega$, $R_{1}=R_{4}=680\;k\Omega$, C=1pF, gain ≈309×): blade-passage pulses are clearly resolved with improved SNR, at the cost of reduced bandwidth.

A further increase to $R_{1}=R_{4}=680\;k\Omega$ yields a voltage gain of approximately 309× and reduces the cutoff frequency to 92.5kHz. The resulting waveform, shown in Figure 14, exhibits clearly resolved blade-passage pulses with a substantially improved SNR relative to the two previous configurations.

The three configurations collectively map the gain–bandwidth trade-off space of the AMR-based conditioning circuit. Table 2 summaries the key parameters across configurations.

The results confirm that the blade-passage signal is detectable by the AMR sensor under the available magnetic bias conditions, but only when sufficient closed-loop gain is applied. The gain required for reliable detection (≥45×) reduces the −3dB bandwidth to levels incompatible with aeronautical turbomachinery BTT at operational rotational speeds. These findings quantify the design constraints for future implementations: achieving both the SNR margin and the bandwidth required for operational BTT will necessitate either an enhanced magnetic bias field to increase the sensor output amplitude, a multi-stage amplification architecture that decouples gain from bandwidth, or sensor-level optimization to reduce the noise floor.

## 6. Conclusions

Blade Tip-Timing (BTT) is among the most promising non-intrusive methodologies for turbomachinery blade vibration measurement, yet its industrial adoption remains constrained by the lack of consolidated calibration procedures, traceable uncertainty quantification, and standardized metrological protocols. This work investigated the feasibility of anisotropic magnetoresistive (AMR) sensors as an alternative transduction technology for BTT, developing a model-based analytical framework for their dynamic characterization and implementing a dedicated signal conditioning circuit.

The analytical model accurately captures the closed-loop gain–bandwidth behavior of the MISO conditioning circuit and proved to be a reliable design tool for parameter selection prior to experimental testing. Once extended to incorporate the parasitic capacitances responsible for the observed discrepancy between predicted and measured cutoff frequencies, it will provide quantitatively accurate estimates across the full operating range. Quantifying these parasitics through dedicated impedance measurements or layout-extracted simulation remains an open item and is planned as part of the next design iteration. The BTT test cases confirm that the AMR sensor responds to blade-induced magnetic perturbations, validating the measurement principle. However, the signal amplitude at the sensor output is constrained by the combined effect of the intrinsic MR ratio of the HMC1501-TR (Honeywell, Morristown, NJ, USA) (≈2.43%), the bias field intensity at the sensor location, and the blade-induced magnetic perturbation at the operating air gap. Under the present geometric configuration, this amplitude is insufficient to resolve blade-passage events without severely compromising bandwidth. As shown in Table 2, reliable detection required gains of 45× or 309×, reducing the bandwidth to levels well below aeronautical BTT requirements, while the only configuration providing adequate bandwidth fails to lift the signal above the noise floor (Figure 12).

Future work should pursue higher-sensitivity Tunnel Magnetoresistance (TMR) sensors as a direct replacement within the same conditioning framework, alongside a systematic optimization of bias magnet geometry, magnet–sensor distance, and blade–sensor air gap, all of which directly govern the field perturbation at the sensing element and could substantially reduce the amplification burden on the conditioning circuit.

## Figures and Tables

**Table 1:** Theoretical and measured −3dB cutoff frequencies for the three conditioning circuit configurations.

$R_{1}=R_{4}$	Theoretical $f_{c}$	Measured $f_{c}$	Ratio
4.6kΩ	33MHz	8.2MHz	0.25
100kΩ	1.5MHz	0.75MHz	0.50
680kΩ	225kHz	92.5kHz	0.41

**Table 2:** Summary of conditioning circuit configurations and BTT detection performance.

Configuration	$R_{1}=R_{4}$	DC Gain	$f_{c}$ (Measured)	Detection
Low gain	4.6kΩ	∼2.1×	8.2MHz	Not detected
Medium gain	100kΩ	∼45×	0.75MHz	Detectable
High gain	680kΩ	∼309×	92.5kHz	Clearly resolved

## Data Availability

The data presented in this study are available on request from the corresponding author.
